# Supplementary material for: Fibrocystin/Polyductin releases a C-terminal fragment that translocates into mitochondria and suppresses cystogenesis
Source: Nat Commun. 2023 Oct 16;14:6513. doi: 10.1038/s41467-023-42196-4 (PMC10579373; doi:10.1038/s41467-023-42196-4)
Supplement: Supplementary file 1 — Supplementary Information [file 41467_2023_42196_MOESM1_ESM.pdf]

Supplementary Figure 1

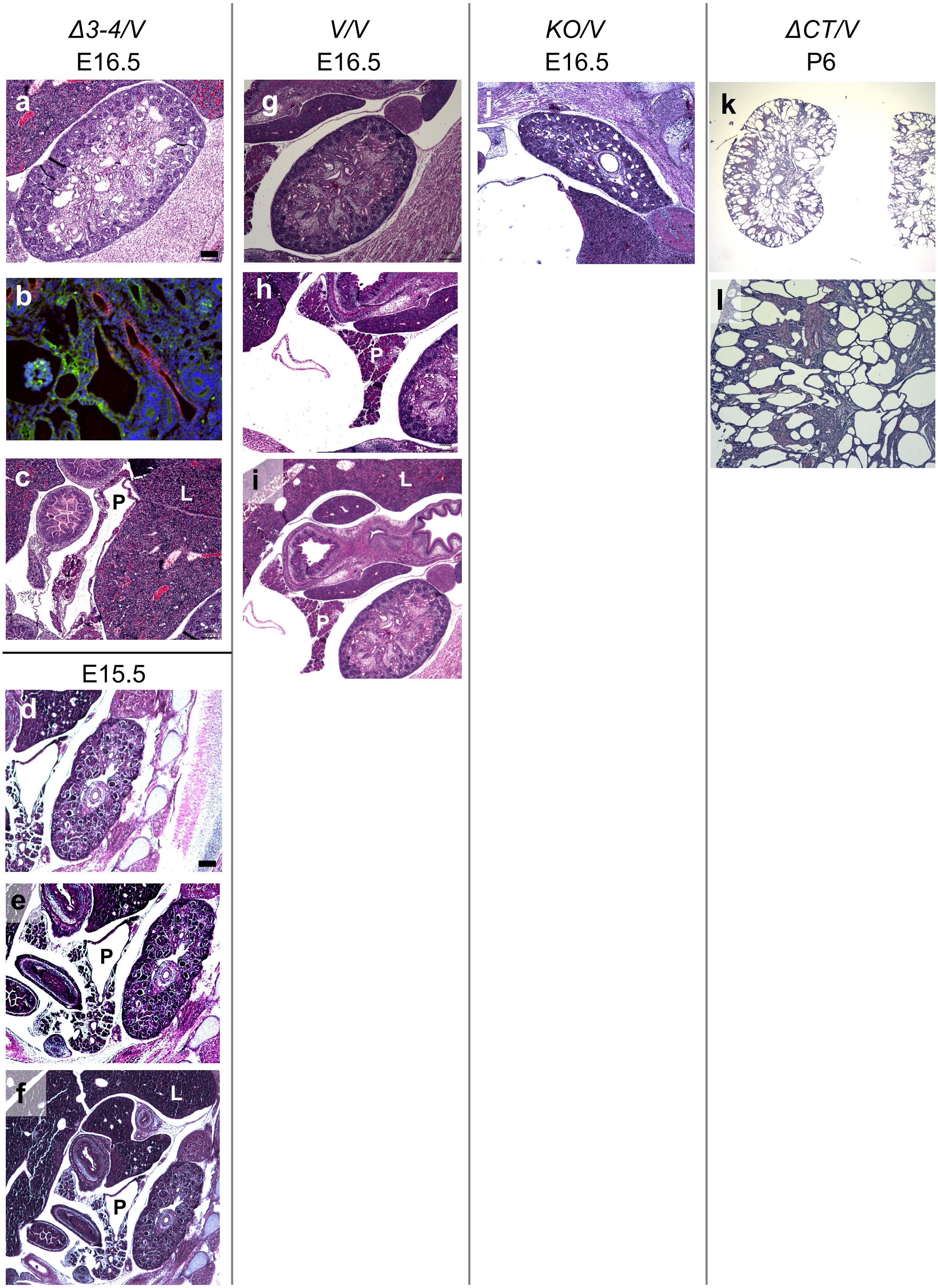

Supplementary Fig. 1: Impact of *Pkhd1* mutations on the kidney and pancreas in *Pkd1*$^{V/V}$ mutants during earlier developmental stages.

Hematoxylin and eosin (H&E), and lectin staining of representative kidney, pancreas, and liver sections. **a** H&E *Δ3-4/V* (*Pkhd1*$^{Δ3\text{-}4/Δ3\text{-}4}$*;Pkd1*$^{V/V}$) kidney at E16.5, scale bar 100 µm. **b** Lectin staining of *Δ3-4/V* kidney at E16.5. Proximal tubule marked by *Lotus tetragonolobus* lectin (LTL)-green, distal tubule/collecting duct marked by *Dolichos biflorus* agglutinin (DBA)-red. **c** H&E *Δ3-4/V* pancreas (P) and liver (L) at E16.5. **d** *Δ3-4/V* kidney at E15.5, scale bar 100 µm. **e** H&E of *Δ3-4/V* pancreas at E15.5. **f** *Δ3-4/V* liver, pancreas, and kidney at E15.5. **g** H&E *V/V* (*Pkd1*$^{V/V}$) kidneys at E16.5. **h** *V/V* pancreas at E16.5. **i** *V/V* liver, pancreas and kidney at E16.5. **j** *KO/V* (*Pkhd1*$^{-/-}$*;Pkd1*$^{V/V}$) kidney at E16.5. **k, l** H&E *ΔCT/V* (*Pkhd1*$^{Δ67/Δ67}$*;Pkd1*$^{V/V}$) kidneys at P6.
