# Supplementary material for: Fibrocystin/Polyductin releases a C-terminal fragment that translocates into mitochondria and suppresses cystogenesis
Source: Nat Commun. 2023 Oct 16;14:6513. doi: 10.1038/s41467-023-42196-4 (PMC10579373; doi:10.1038/s41467-023-42196-4)
Supplement: Supplementary file 3 — Reporting Summary [file 41467_2023_42196_MOESM3_ESM.pdf]

nature portfolio

Corresponding author(s): Feng Qian

Last updated by author(s): Sep 12, 2013

Reporting Summary

Nature Portfolio wishes to improve the reproducibility of the work that we publish. This form provides structure for consistency and transparency in reporting. For further information on Nature Portfolio policies, see our Editorial Policies and the Editorial Policy Checklist.

Please do not complete any field with "not applicable" or n/a. Refer to the help text for what text to use if an item is not relevant to your study.
For final submission: please carefully check your responses for accuracy; you will not be able to make changes later.

Statistics

For all statistical analyses, confirm that the following items are present in the figure legend, table legend, main text, or Methods section.

n/a	Confirmed	
☐	☒	The exact sample size (*n*) for each experimental group/condition, given as a discrete number and unit of measurement
☐	☒	A statement on whether measurements were taken from distinct samples or whether the same sample was measured repeatedly
☐	☒	The statistical test(s) used AND whether they are one- or two-sided<br>*Only common tests should be described solely by name; describe more complex techniques in the Methods section.*
☒	☐	A description of all covariates tested
☒	☐	A description of any assumptions or corrections, such as tests of normality and adjustment for multiple comparisons
☐	☒	A full description of the statistical parameters including central tendency (e.g. means) or other basic estimates (e.g. regression coefficient) AND variation (e.g. standard deviation) or associated estimates of uncertainty (e.g. confidence intervals)
☐	☒	For null hypothesis testing, the test statistic (e.g. *F*, *t*, *r*) with confidence intervals, effect sizes, degrees of freedom and *P* value noted<br>*Give P values as exact values whenever suitable.*
☒	☐	For Bayesian analysis, information on the choice of priors and Markov chain Monte Carlo settings
☒	☐	For hierarchical and complex designs, identification of the appropriate level for tests and full reporting of outcomes
☒	☐	Estimates of effect sizes (e.g. Cohen's *d*, Pearson's *r*), indicating how they were calculated

Our web collection on statistics for biologists contains articles on many of the points above.

Software and code

Policy information about availability of computer code

Data collection	All software we use in the manuscript are described in published literature. Fluorescent images were obtained using Zeiss Axio Observer D1 microscope and the associated software ZEN (blue edition). Whole kidney images from hematoxylin and eosin–stained sagittal kidney sections were obtained at a 1.6x and 20x magnification using Nikon Eclipse E600 microscope and the associated software Lumenera Infinity Analyze. Immunoblotting images were obtained using a BioRad ChemiDoc Imaging System and the associated software of Image Lab Software Version 6.1, or developed on film.
Data analysis	Data analysis was undertaken using Microsoft Excel (office 365), R studio (version 1.4.1106) (https://www.rstudio.com/_). Mitochondria shape descriptors and cystic areas were calculated using ImageJ software (https://imagej.nih.gov/ij/, NIH) (version 1.54f). Mitochondrial localization signal prediction was undertaken using Mitoprot II (https://ihg.helmholtz-muenchen.de/ihg/mitoprot.html).

For manuscripts utilizing custom algorithms or software that are central to the research but not yet described in published literature, software must be made available to editors and reviewers. We strongly encourage code deposition in a community repository (e.g. GitHub). See the Nature Portfolio guidelines for submitting code & software for further information.

Data

Policy information about availability of data

All manuscripts must include a data availability statement. This statement should provide the following information, where applicable:

- Accession codes, unique identifiers, or web links for publicly available datasets
- A description of any restrictions on data availability
- For clinical datasets or third party data, please ensure that the statement adheres to our policy

All data generated or analysed during this study are included in this paper (and its supplementary information files). Source data are provided with this paper. Full-length immunoblots are provided in the Source Data File.

Research involving human participants, their data, or biological material

Policy information about studies with human participants or human data. See also policy information about sex, gender (identity/presentation), and sexual orientation and race, ethnicity and racism.

Reporting on sex and gender	This study did not involve human participants, their data, or biological material
Reporting on race, ethnicity, or other socially relevant groupings	This study did not involve human participants, their data, or biological material
Population characteristics	This study did not involve human participants, their data, or biological material
Recruitment	This study did not involve human participants, their data, or biological material
Ethics oversight	This study did not involve human participants, their data, or biological material

Note that full information on the approval of the study protocol must also be provided in the manuscript.

Field-specific reporting

Please select the one below that is the best fit for your research. If you are not sure, read the appropriate sections before making your selection.

☒ Life sciences ☐ Behavioural & social sciences ☐ Ecological, evolutionary & environmental sciences

Life sciences study design

All studies must disclose on these points even when the disclosure is negative.

Sample size	Sample sizes for experiments involving Pkd1 and Pkhd1 mutant mice were chosen based on previous analyses with similar experimental animal system (Hyunho Kim, Hangxue Xu, Qin Yao, Weizhe Li, Qiong Huang, Patricia Outeda, Valeriu Cebotaru, Marco Chiaravalli, Alessandra Boletta, Klaus Piontek, Gregory G. Germino, Edward J. Weinman, Terry Watnick & Feng Qian. Ciliary membrane proteins traffic through the Golgi via a Rabep1/GGA1/Arl3-dependent mechanism. Nat Commun 5, 5482 (2014).<br>For non-animal experiments, sample sizes were chosen based on our previous experience with the relevant assay systems.
Data exclusions	No data were excluded.
Replication	To ensure robust reproducibility: All data presented in this manuscript were repeated at least three times. All attempts at replication were successful. All immunofluorescence, immunoblot, and TEM assays were repeated at least three times. All confocal images presented were imaged at least thrice for a single data point. For transgenic mouse lines: at least three individual animals for each genotype were analyzed. For quantification of cystic area calculation used in the manuscript, three independent replicates were used for each genotype tested. Results from technical and biological replicates were consistent among them.
Randomization	The allocation of animals in each group was based exclusively on genotype without any exclusion. For all non-animal experiments, in-vitro samples were randomly assigned to the relevant experiment protocols.
Blinding	The investigators were blinded to group allocation during data collection and analysis. For example, Images were blinded to genotype and processed for shape descriptor analysis and cristae diameter measurements.

Reporting for specific materials, systems and methods

We require information from authors about some types of materials, experimental systems and methods used in many studies. Here, indicate whether each material, system or method listed is relevant to your study. If you are not sure if a list item applies to your research, read the appropriate section before selecting a response.

Materials & experimental systems

n/a	Involved in the study
☐	☒ Antibodies
☐	☒ Eukaryotic cell lines
☒	☐ Palaeontology and archaeology
☐	☒ Animals and other organisms
☒	☐ Clinical data
☒	☐ Dual use research of concern
☒	☐ Plants

Methods

n/a	Involved in the study
☒	☐ ChIP-seq
☒	☐ Flow cytometry
☒	☐ MRI-based neuroimaging

Antibodies

Antibodies used

1) Rat monoclonal anti-FPC E3 (home-made); Immunoblot, 1:500-1000 dilution.
2) Rat monoclonal anti-FPC E4 (home-made); Immunoblot, 1:500-1000 dilution.
3) Rat monoclonal anti-FPC E1 (home-made); Immunoblot, 1:500-1000 dilution.
4) Rat monoclonal anti-PC1 E8 (home-made); Immunoblot, 1:500-1000 dilution.
5) Mouse monoclonal anti-Pyruvate Dehydrogenase (Abcam, 13G2AE2BH5); Immunoblot at 1 µg/ml.
6) Rabbit monoclonal anti-APN (Abcam, ab108310); Immunoblot, 1:1000 dilution.
7) Rabbit polyclonal anti-THP, Tamm-Horsfall glycoprotein/Uromodulin (Santa Cruz, sc-20631); Immunoblot, 1:1000 dilution.
8) Rabbit polyclonal anti-Aquaporin2, AQP2, (Sigma, A7310); Immunoblot, 1:200-1:1000 dilution.
9) Mouse monoclonal anti-beta-Actin (Sigma, A5441); Immunoblot, 1:5000-10,000 dilution.
10) Rabbit monoclonal anti-E-cadherin, E-Cad (Cell Signaling, 3195P); Immunoblot, 1:1000 dilution.
11) Rabbit monoclonal anti-TOM20 (Cell Signaling, 42406T); Immunoblot, 1:500-1000 dilution.
12) Mouse monoclonal anti-Tubulin (Sigma, T6793); Immunoblot, 1:2000 dilution.
13) Mouse monoclonal Actin-Rhodamine (BioRad, 12004163); Immunoblot, 1:1000-10,000 dilution.
14) Goat anti-Rat HRP (Sigma, NA935V); Immunoblot, 1:1000-10,000 dilution.
15) Alexa Fluor donkey anti-mouse 555 (Invitrogen, A31570); Immunoblot, 1:1000-10,000 dilution.
16) Alexa Fluor donkey anti-rabbit 647 (Invitrogen, A31573); Immunoblot, 1:1000-10,000 dilution.
17) Lotus Tetragonolobus Lectin (LTL), Fluorescein (Vector Laboratories, FL-1321-2); Immunofluorescence at 2 mg active conjugate/ml.
18) Dolichos Biflorus Agglutinin (DBA), Rhodamine (Vector Laboratories, RL-1032-2); Immunofluorescence at 2 mg active conjugate/ml.

Validation

Our home-made primary antibodies used in this study have been validated using mouse tissues and cells that are genetically null. Other primary antibodies used in this study have been validated by manufacturer and/or in literature.

1) this study (Fig 1g)
2) this study (Fig 1g)
3) https://pubmed.ncbi.nlm.nih.gov/28729032/ and this study (Fig 1g)
4) https://pubmed.ncbi.nlm.nih.gov/24958103/ and this study (Fig 5j and k)
5) https://www.abcam.com/pyruvate-dehydrogenase-e2e3bp-antibody-13g2ae2bh5-ab110333.html. DOI: 10.15252/embj.2020105268.
6) https://www.abcam.com/products/primary-antibodies/cd13-antibody-epr4058-ab108310.html. doi: 10.1186/s12958-015-0088-y
7) https://www.scbt.com/p/thp-antibody-h-135?requestFrom=search. DOI: 10.1038/srep42970
8) https://www.sigmaaldrich.com/US/en/product/sigma/a7310. DOI: 10.1007/s00418-008-0457-0
9) https://www.sigmaaldrich.com/US/en/product/sigma/a5441. DOI: 10.1038/s41467-019-11614-x
10)https://www.cellsignal.com/products/primary-antibodies/e-cadherin-24e10-rabbit-mab/3195?_requestid=873769. DOI: 10.1038/s41598-023-36451-3.
11)https://www.cellsignal.com/products/primary-antibodies/tom20-d8t4n-rabbit-mab/42406. DOI: 10.1038/s41467-022-34632-8
12)https://www.sigmaaldrich.com/US/en/product/sigma/t6793. DOI: 10.1242/dev.170241
13)https://www.bio-rad.com/en-us/sku/12004163-hfab-rhodamine-anti-actin-primary-antibody-200-ul?ID=12004163
14)https://www.sigmaaldrich.com/US/en/product/sigma/gena935
15)https://www.thermofisher.com/antibody/product/Donkey-anti-Mouse-IgG-H-L-Highly-Cross-Adsorbed-Secondary-Antibody-Polyclonal/A-31570
16)https://www.thermofisher.com/antibody/product/Donkey-anti-Rabbit-IgG-H-L-Highly-Cross-Adsorbed-Secondary-Antibody-Polyclonal/A-31573. https://www.nature.com/articles/s41467-023-41037-8
17) https://vectorlabs.com/products/fluorescein-lotus-tetragonolobus-lectin-ltl. DOI: 10.1073/pnas.0708217104
18)https://vectorlabs.com/products/rhodamine-dolichos-biflorus-agglutinin-dba. DOI: 10.1073/pnas.0708217104

Eukaryotic cell lines

Policy information about cell lines and Sex and Gender in Research

Cell line source(s)

HEK293 (ATCC, CRL-1573); MDCK; mIMCD3 (ATCC; CRL-2123)

Authentication	Cell morphology aligned with cell type and was approved by experienced cell culturists.
Mycoplasma contamination	All cell lines were tested to be free of mycoplasma contamination.
Commonly misidentified lines (See ICLAC register)	None of the used cell lines is listed in ICLAC database.

Animals and other research organisms

Policy information about studies involving animals; ARRIVE guidelines recommended for reporting animal research, and Sex and Gender in Research

Laboratory animals	Details on the mice used in this study have been included in this manuscript. In this study, we used various PKD models, with various combinations of Pkd1 and Pkhd1 mutant alleles Pkd1V/V, Pkhd1Flox67HA/Flox67HA, Pkhd1LSL/LSL, Pkhd1D3-4/D3-4 or Pkhd1D67/D67, at embryonic stages and pups. Animal studies were performed in adherence to the NIH Guide for the Care and Use of Laboratory Animals and approved by the University of Maryland School of Medicine Institutional Animal Care and Use Committee. Both male and female mice were used for experiments since phenotypes were consistent between sexes. Littermates were compared when possible. All mice were congenic on a C57BL/6J background.
Wild animals	No wild animals were used in this study.
Reporting on sex	Both male and female mice were used for experiments since phenotypes were consistent between sexes.
Field-collected samples	No field-collected samples were used in this study.
Ethics oversight	Animal studies were performed in adherence to the NIH Guide for the Care and Use of Laboratory Animals and approved by the University of Maryland School of Medicine Institutional Animal Care and Use Committee (Protocol # 0421008).

Note that full information on the approval of the study protocol must also be provided in the manuscript.

This checklist template is licensed under a Creative Commons Attribution 4.0 International License, which permits use, sharing, adaptation, distribution and reproduction in any medium or format, as long as you give appropriate credit to the original author(s) and the source, provide a link to the Creative Commons license, and indicate if changes were made. The images or other third party material in this article are included in the article's Creative Commons license, unless indicated otherwise in a credit line to the material. If material is not included in the article's Creative Commons license and your intended use is not permitted by statutory regulation or exceeds the permitted use, you will need to obtain permission directly from the copyright holder. To view a copy of this license, visit http://creativecommons.org/licenses/by/4.0/
